# Severe Apathy Due to Injury of Prefronto-caudate Tract

**DOI:** 10.1515/tnsci-2019-0027

**Published:** 2019-07-22

**Authors:** Sung Ho Jang, Hyeok Gyu Kwon

**Affiliations:** 1 Department of Physical Medicine and Rehabilitation, College of Medicine, Yeungnam University, Gyeongsan, South Korea; 2 Department of Physical Therapy, College of Health Science, Eulji University, Daejeon, South Korea

**Keywords:** apathy, prefrontal cortex, caudate nucleus, hypoxic–ischemic brain injury, diffusion tensor tractography

## Abstract

The caudate nucleus, which is vulnerable to hypoxic–ischemic brain injury (HI-BI), is important to cognitive function because it is connected to the prefrontal cortex. Using diffusion tensor tractography (DTT), no study on injury of the prefronto-caudate tract in a patient with HI-BI has been reported so far. Here, we report a patient with severe apathy who showed injury of the prefronto-caudate tract following HI-BI, which was demonstrated by DTT. A 38-year-old female patient suffered HI-BI induced by carbon monoxide poisoning following attempted suicide for a period of approximately four hours. From the onset, the patient showed severe apathy (7 months after onset-the Apathy Scale score was 24 [full score: 42]). Brain MR images taken at seven months after onset showed no abnormality. On 7-month DTT, the neural connectivity of the caudate nucleus to the medial prefrontal cortex (Brodmann area: 10 and 12) and orbitofrontal cortex (Brodmann area: 11 and 13) was decreased in both hemispheres. Using DTT, injury of the prefronto-caudate tract was demonstrated in a patient who showed severe apathy following HI-BI. We believe that injury of the prefronto-caudate tract might be a pathogenetic mechanism of apathy in patients with HI-BI.

## Introduction

Hypoxic–ischemic brain injury (HI-BI), caused by insufficient oxygen supply to the brain because of various etiologies including cardiopulmonary arrest, respiratory failure, or carbon monoxide poisoning, affects various brain regions [1]. In particular, several specific brain regions including the basal ganglia, hippocampus, cerebellum, and thalamic nuclei are known to be vulnerable to HI-BI [2]. Among these vulnerable brain regions, the caudate nucleus (CN), a part of the basal ganglia, is important for cognitive function by connecting to the prefrontal cortex. Thus, the prefronto-caudate tract which is known to be related to apathy, anxiety, depression, and disinhibition, might be vulnerable to HI-BI [3]. In particular, injury of the prefronto-caudate tract, which is connected to the medial prefrontal cortex (PFC), is associated with apathy [4].

Diffusion tensor tractography (DTT) derived from diffusion tensor imaging (DTI) has an advantage in estimating and visualizing the connectivity of the CN to the PFC and enables detection of subtle or invisible neural injury by detection of characteristics of water diffusion [5]. In particular, probabilistic DTT, which considers the distribution of underlying fiber structure, has been widely used for investigation of the neural connectivity of neural structures in the human brain [5]. As a result, several studies have investigated the frontostriatal tract including the prefronto-caudate tract in patients with obsessive-compulsive disorder, bipolar mood disorder, attention-deficit/hyperactivity disorder, and schizophrenia [6]. However, no study using DTT to investigate injury of the prefronto-caudate tract in patients with HI-BI has been reported so far. We hypothesized that DTT could demonstrate injury of the prefronto-caudate tract in a patient who showed severe apathy even though conventional brain MRI did not reveal any specific brain lesion.

In this study, we report a patient who showed injury of the prefronto-caudate tract following HI-BI, which was demonstrated by DTT.

## Case Report

**Fig. 1:**
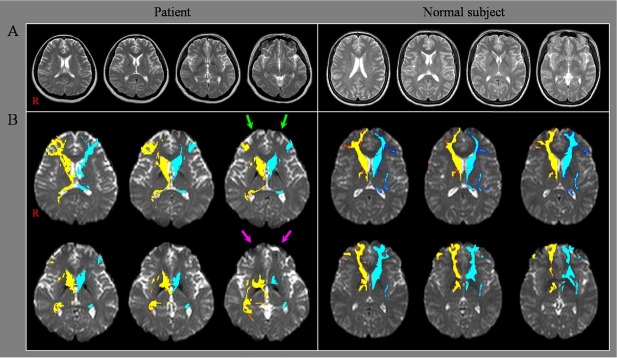
(A) T2-weighted brain MR images at seven months after onset show no abnormality. (B) The neural connectivities of the caudate nucleus to Brodmann areas 10 and 12 (green arrows, medial prefrontal cortex) and Brodmann areas 11 and 13 (purple arrows, orbitofrontal cortex) in both hemispheres are decreased compared to a normal subject (40 year-old female).

A 38-year-old female patient was diagnosed with depression and received medication. Her depression was improved with medication; however, after stopping medication, her depression was worsened. One year after stopping her medication, she suffered HI-BI induced by carbon monoxide poisoning following attempted suicide for a period of approximately four hours and was immediately transferred to the emergency room of a local medical center. At seven months after onset, she was admitted to the department of rehabilitation of a university hospital for rehabilitation. Her mini-mental state examination resulted in a score of 28 (orientation: -2). The patient showed mainly severe apathy with no spontaneous speech, activity, or hypersomnolence (the Apathy Scale score: 24 [full score: 42 score]), depression and gait disturbance (Table 1). She underwent rehabilitative management and administration of drugs (Pramipexole: 0.375mg, Venlafaxine HCI: 225mg, Mirtazapine: 30mg, Aripiprazole: 10mg) Brain MR images taken at seven months after onset showed no abnormality (Fig.1-A). The patient’s husband provided signed, informed consent, and our institutional review board approved the study protocol.

**Table 1:** Apathy scale of the patient

No	Question	Score
1	Are you interested in learning new things?	2
2	Does anything interest you?	2
3	Are you concerned about your condition?	1
4	Do you put much effort into things?	2
5	Are you always looking for something to do?	2
6	Do you have plans and goals for the future?	2
7	Do you have motivation?	2
8	Do you have the energy for daily activities?	2
9	Does someone have to tell you what to do each day?	2
10	Are you indifferent to things?	2
11	Are you unconcerned with many things?	1
12	Do you need a push to get started on things?	2
13	Are you neither happy nor sad, just in between?	1
14	Would you consider yourself apathetic? Total	1
	Total	24

questions 1-8: not at all = 3, slightly = 2, some = 1, a lot = 0 questions 9-14: not at all = 0, slightly = 1, some = 2, a lot = 3

DTI data was acquired at seven months after onset using a 6-channel head coil on a 1.5T Philips Gyroscan Intera (Philips, Ltd., Best, the Netherlands) with 32 diffusion gradients by single-shot echo-planar imaging. The imaging parameters were as follows: acquisition matrix = 96×96; reconstructed to matrix = 192×192; field of view = 240×240mm^2^; TR = 10,398ms; TE = 72ms; parallel imaging reduction factor = 2; echo-planar imaging factor= 59; b= 1000s/mm^2^; and slice thickness of 2.5mm. Head motion effect and image distortion due to eddy current were corrected by affine multi-scale two-dimensional registration. Fiber tracking was performed using probabilistic tractography, and applied in the default tractography option in the Oxford Centre for Functional Magnetic Resonance Imaging of the Brain (FMRIB) Diffusion Software (5000 streamline samples, 0.5 mm step lengths, curvature thresholds = 0.2). This fiber tracking method calculated and generated 5000 streamline samples from seed regions of interest with reflection of both dominant and non-dominant orientation of diffusion in each voxel and showed how they connect the brain regions. For the connectivity of the CN, the seed region of interest was placed on the CN which was isolated by adjacent structures (medial boundary: lateral ventricle, lateral boundary: the anterior limb of the internal capsule). The threshold of 10 streamlines was applied for the results of fiber tracking.

The neural connectivity of the CN to the medial PFC (Brodmann area [BA]: 10 and 12) and orbitofrontal cortex (BA: 11 and 13) was decreased in both hemispheres.

## Discussion

In the current study, we investigated the neural connectivity of the CN in a patient with HI-BI using DTT and found that the neural connectivity of the CN to the medial PFC (BA 10 and 12) and orbitofrontal cortex (BA 11 and 13) was decreased in both hemispheres. This decreased neural connectivity appeared to indicate injury of the prefronto-caudate tract. Because no definite brain lesion was observed on conventional brain MRI, HI-BI was the most plausible pathogenetic mechanism for injury of the prefronto-caudate tract. As a result, the patient’s severe apathy appeared to be ascribed, at least in part, to injury of the prefronto-caudate tract. Therefore, our results suggest the necessity of evaluation of the prefronto-caudate tract in patients who show apathy after HI-BI, even though no definite brain lesion on conventional brain MRI was observed.

Many studies have reported injury of the uni- or bilateral CN in patients with apathy, using conventional MRI [7,8]; however, only a few studies have reported such injuries in the prefronto-caudate tract [6,9,10]. In 2005, Valente et al. found abnormalities of the frontal-striatal-thalamic circuit including CN in 19 patients with obsessive-compulsive disorder using a voxel-based morphometry [6]. The next year, Rose et al. reported injury of the prefrontal-thalamic circuitry including CN in 12 patients with schizophrenia using DTI [9]. Subsequently, Casey et al. (2007) found an association of connectivity of the frontal cortex with the CN with cognitive control in 20 parent-child dyads with attention-deficit/hyperactivity disorder using DTI [10]. Consequently, to the best of our knowledge, this is the first study to demonstrate injury of the prefronto-caudate tract in patients with HI-BI.

In conclusion, using DTT, injury of the prefronto-caudate tract was demonstrated in a patient who showed severe apathy following HI-BI. We believe that injury of the prefronto-caudate tract might be a pathogenetic mechanism of apathy in patients with HI-BI. It should be noted that this study is limited because it is based on a case report. When using DTT, both false positive and negative results can occur throughout the white matter of the brain because of complex fiber configurations such as crossing or kissing fibers and partial volume effects. Therefore, we suggest further studies involving larger numbers of patients be conducted to overcome the limitations of DTI.
